# Anorexia nervosa and inflammatory bowel diseases—Diagnostic and genetic associations

**DOI:** 10.1002/jcv2.12036

**Published:** 2021-09-27

**Authors:** Janne Tidselbak Larsen, Zeynep Yilmaz, Bjarni Jóhann Vilhjálmsson, Laura M. Thornton, Michael Eriksen Benros, Katherine L. Musliner, Eating Disorders Working Group of the Psychiatric Genomics Consortium, Thomas Werge, David M. Hougaard, Preben Bo Mortensen, Cynthia M. Bulik, Liselotte Vogdrup Petersen

**Affiliations:** ^1^ National Centre for Register‐based Research Aarhus BSS Aarhus University Aarhus Denmark; ^2^ Lundbeck Foundation Initiative for Integrative Psychiatric Research (iPSYCH) Aarhus University Aarhus Denmark; ^3^ Centre for Integrated Register‐based Research (CIRRAU) Aarhus University Aarhus Denmark; ^4^ Department of Psychiatry University of North Carolina at Chapel Hill Chapel Hill North Carolina USA; ^5^ Department of Genetics University of North Carolina at Chapel Hill Chapel Hill North Carolina USA; ^6^ Copenhagen Research Centre for Mental Health Mental Health Centre Copenhagen Copenhagen University Hospital Hellerup Denmark; ^7^ Research Institute of Biological Psychiatry Mental Health Center Sanct Hans Copenhagen University Hospital Roskilde Denmark; ^8^ Department of Clinical Medicine University of Copenhagen Copenhagen Denmark; ^9^ Danish Center for Neonatal Screening Department of Congenital Disorders Statens Serum Institut Copenhagen Denmark; ^10^ Department of Nutrition University of North Carolina at Chapel Hill Chapel Hill North Carolina USA; ^11^ Department of Medical Epidemiology and Biostatistics Karolinska Institutet Stockholm Sweden

**Keywords:** anorexia nervosa, Crohn's disease, eating disorders, inflammatory bowel diseases, ulcerative colitis

## Abstract

**Background:**

Anorexia nervosa (AN), a serious eating disorder, and inflammatory bowel diseases (IBD) share a number of key symptoms, for example, discomfort during eating and early satiety. Despite the symptom overlap, studies on comorbidity are limited and mostly conducted in relatively small samples. This study investigates the comorbidity of diagnosed AN with IBD, and the subtypes Crohn's disease and ulcerative colitis, in a population‐based sample and explores whether genetic factors could play a role in the overlap.

**Methods:**

The study included 1,238,813 individuals born in Denmark 1981–2005 selected from the population register (5067 diagnosed with AN and 6947 diagnosed with any IBD), including a subsample of 23,236 individuals with genetic information (4271 with AN and 176 with any IBD). By combining hospital‐based diagnoses recorded in health registers until 2013 with polygenic scores (PGS) of AN and IBD, we investigated possible associations between diagnoses of each disorder, both within individuals and families, and between PGS of one disorder and diagnosis of the other disorder. Analyses were conducted using Cox regression and logistic regression.

**Results:**

We found that a prior diagnosis of AN was associated with hazard ratios of 1.44 (1.05, 1.97) for any IBD, 1.60 (1.04, 2.46) for Crohn's disease, and 1.66 (1.15, 2.39) for ulcerative colitis, whereas IBD diagnoses were not significantly associated with later AN diagnosis. No significant within‐families associations were observed. We found no associations between AN and IBD using PGS.

**Conclusions:**

AN was associated with later risk of IBD, Crohn's disease, and ulcerative colitis; however, the reverse was not observed. It is important for clinicians to be aware of this association to evaluate IBD as a differential diagnosis or an emergent condition in patients with AN.

## Graphical abstract

Despite an overlap in symptoms, research on the comorbidity between anorexia nervosa and inflammatory bowel diseases is limited.This study examines the associations between anorexia nervosa and inflammatory bowel disease in both directions in a large, population‐based sample and finds that prior diagnoses of anorexia nervosa are associated with higher rates of later inflammatory bowel disease

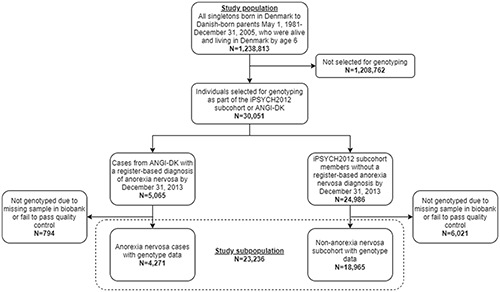

## INTRODUCTION

Anorexia nervosa (AN) is characterized by an intense fear of weight gain and low body mass index (BMI), sustained through food restriction, excessive energy expenditure, and/or purging behavior. Digestive symptoms and gastrointestinal complications are common in AN (Hetterich et al., 2019; Norris et al., 2016; Salvioli et al., 2013; Schalla & Stengel, 2019; Zipfel et al., 2006).

**Key points**

- Despite sharing some key symptoms, research on the comorbidity between anorexia nervosa (AN) and inflammatory bowel diseases is limited and based on smaller samples
- Based on a large, population‐based sample, we examined the associations between diagnosed AN and inflammatory bowel disease and between genetic liability captured using polygenic scores and disease risk in both directions
- We found that prior diagnoses of AN were associated with higher rates of later inflammatory bowel disease, whereas we found no evidence of an association in the opposite direction
- Clinicians treating patients with AN should be aware of inflammatory bowel disease as a potential comorbidity

Inflammatory bowel diseases (IBD) are a group of conditions characterized by chronic inflammation of the digestive tract, with the two most common IBD subtypes being Crohn's disease and ulcerative colitis. IBD can result in pain or nausea during eating and/or early satiety. Thus, patients with IBD may avoid certain foods they experience to trigger their symptoms, potentially leading to altered eating behavior and weight loss (Hughes et al., 2016).

Despite the observable symptom overlap between AN and IBD, comorbidity between these disorders has not been studied extensively. A 2014 Finnish study reported an increased risk of prior gastroenterological immune‐mediated diseases, including Crohn's disease and ulcerative colitis, in AN patients compared with controls. They did not find significantly increased rates of gastroenterological diseases following AN onset (Raevuori et al., 2014). A study based in the UK Record Linkage Cohort Study reported increased risk of both Crohn's disease following prior AN and of AN following prior Crohn's disease (Wotton et al., 2016). A systematic review by Ilzarbe et al. based on 14 articles, encompassing only 219 cases in total (Ilzarbe et al., 2017) found that in cases diagnosed with both types of disorders, 50% were first diagnosed with an eating disorder and 50% with an IBD, and that the most frequently reported comorbidity was AN and Crohn's disease (*n* = 90). Comorbid diagnoses of AN and IBD can lead to challenges in both diagnosis and treatment, with misdiagnosis or inappropriate treatment having the potential to exacerbate both illnesses (Mascolo et al., 2017). The mechanisms behind the associations is suggested to include misdiagnosis due to common symptoms, a causal relationship where one disorder leads to the other, or shared environmental and/or genetic risk factors (Santonicola et al., 2019). A study of genetic correlations between psychiatric and immune‐related phenotypes identified no significant correlations for AN with either Crohn's disease or ulcerative colitis (Tylee et al., 2018).

The purpose of this study was to investigate to what degree AN and IBD are associated in a large population‐based cohort and whether genetic associations could explain any observed association, which may help identify individuals at high risk and shed light on possible etiological mechanisms. In order to determine whether any potential associations are specific to AN, we conduct similar analyses of associations between IBD and depression for comparison.

## MATERIALS AND METHODS

### Data sources

This study was conducted by combining information from Danish population‐based registers, polygenic scores (PGS) and summary statistics from genome‐wide association studies (GWAS). All Danish citizens are assigned a civil registration number at birth or upon immigration, which is used as a unique identifier in all national registers and enables accurate linkage between them. Information on dates of birth, death, and migration; place of birth; and identity of parents were obtained from the Danish Civil Registration System, established in 1968 (Pedersen, 2011). Information on contacts to hospitals was obtained from the Danish National Patient Register (Lynge et al., 2011) and the Danish Psychiatric Central Research Register (Mors et al., 2011). The National Patient Register and the Psychiatric Central Research Register contain information on all hospital inpatient contacts since their establishment in 1977 and 1969, respectively, and since 1995 also outpatient and emergency room contacts. The diagnostic system used in Denmark was the International Classification of Diseases, 8th revision (ICD‐8) until 1994, when it was replaced by the International Classification of Diseases, 10th revision (ICD‐10). Data on parental educational levels were obtained from the Danish Population Education Register (Jensen & Rasmussen, 2011).

Since May 1981, dried blood spot samples from all nearly all Danish newborns have been collected after routine screening for congenital diseases within the first few days of life and stored at the Danish Newborn Screening Biobank (Nørgaard‐Pedersen & Hougaard, 2007).

This study was approved by the Danish Data Protection Agency and the Danish Scientific Ethics Committee.

### Study population

The various parts of the study were conducted in two study populations, one of which encompassed the other.

The study population used in the register‐only part of the study was defined as singletons born in Denmark between May 1, 1981 and December 31, 2005, who were alive and living in Denmark on their 6th birthday. To ensure complete parental history of psychiatric disorders, only individuals with known and Danish‐born mother and father were retained, resulting in a study population of 1,238,813 individuals (600,774 female, 48.69%).

**FIGURE 1:**
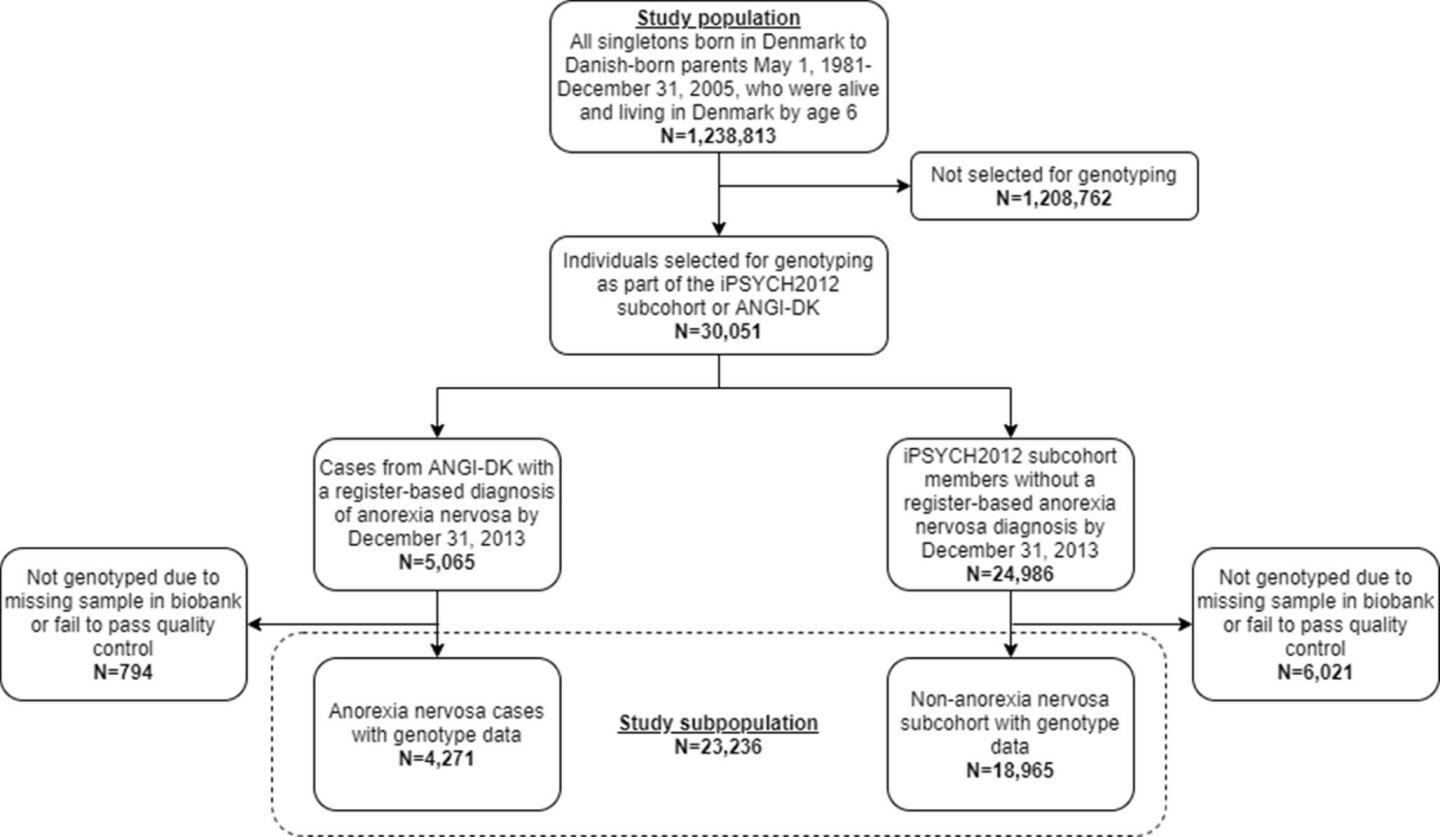
Flowchart depicting the inclusion of individuals in the study population and in the study subpopulation

A study subpopulation was used when combining register information with PGS and comprised individuals from the first study population who either had a register‐based diagnosis of ICD‐10 broad AN (F50.0, F50.1) after their 6th birthday (*N* = 5065) or were included in a subcohort chosen at random from the eligible background population (*N* = 24,986). The AN cases originated from the Danish branch of the Anorexia Nervosa Genetics Initiative (ANGI), described in detail by Thornton et al. (2018), whereas the random subcohort came from the iPSYCH2012 case‐cohort sample, described by Pedersen et al. (2018). Individuals who had not been genotyped due to missing blood spots in the biobank, who were not of European ancestry, or whose genotype data failed to pass quality control were excluded from the study population. In case of relatedness in the sample, one person from each related pair was removed at random. In total, 794 AN cases and 6021 subcohort members were removed, resulting in a final subpopulation of 23,236 individuals (12,808 female, 55.12%), including 4271 AN cases and 18,965 randomly chosen individuals without a registered AN diagnosis. Figure 1 depicts the inclusion criteria for both the larger study population used for register‐only analyses and the smaller study subpopulation used for combined register and genetic data. A subpopulation of 40,438 individuals (23,369 female, 57.79%) was included in the analyses including PGS for the comparison disorder depression, consisting of 18,761 depression cases and 21,677 randomly chosen individuals with no registered diagnosis of depression, both from the iPSYCH2012 study. Figure S1 depicts the populations used for analyses of depression.

### Diagnoses

We included information on the first contact to a hospital resulting in a discharge diagnosis of broad AN (ICD‐10: F50.0, F50.1), Crohn's disease (ICD‐8: 563.0x; ICD‐10: K50), ulcerative colitis (ICD‐8: 563.19, 569.04; ICD‐10: K51), and IBD (Crohn's disease and ulcerative colitis combined; Harpsøe et al., 2014). For the comparison disorder depression, diagnoses of F32 and F33 were included. All contacts were considered except for referral diagnoses, and for AN and depression, only contacts initiated after the 6th birthday were included. The date of onset was defined as the admission date of the first contact with the discharge diagnosis of interest. Parental diagnoses were defined in a similar manner, but also included ICD‐8 diagnoses of AN (306.50) and depression (296.09, 296.29, 298.09, and 300.49). Individuals were considered exposed to parental history of each specific disorder if at least one parent had been diagnosed.

### Summary statistics files and PGS

Inclusion of genetic information was based on genotyping of DNA extracted from neonatal dried blood spot samples stored in the Danish Newborn Screening Biobank. Retrieval of blood samples, genotyping, and quality control have been described in detail elsewhere (Pedersen et al., 2018; Thornton et al., 2018; Watson et al., 2019). Summary statistics files used in the calculation of PGS and for Mendelian randomization (MR) were obtained from genome‐wide association studies (GWAS) of AN (Watson et al. [2019] excluding Danish contributions for PGS calculations), IBD, Crohn's disease, ulcerative colitis (de Lange et al., 2017), and depression excluding Danish samples (Wray et al., 2018). 23andMe is a direct‐to‐consumer provider of genetic testing which also collects phenotype data among its customers using surveys. Participants provided informed consent and participated in the research online, under a protocol approved by the external AAHRPP‐accredited IRB, Ethical & Independent Review Services (E&I Review). The full GWAS summary statistics for the 23andMe discovery data set will be made available through 23andMe to qualified researchers under an agreement with 23andMe that protects the privacy of the 23andMe participants. Please visit https://research.23andme.com/collaborate/#dataset‐access/ for more information and to apply to access the data. PGS are one‐dimensional measures of genetic liability of a specific trait for an individual in a target sample, calculated as a sum of risk variants across the genome, weighted by associated effect sizes derived from a GWAS performed in an independent discovery sample. In this study, PGS were calculated for each individual accounting for linkage disequilibrium (LD) using the LDpred method (Vilhjálmsson et al., 2015) in two batches: (1) the iPSYCH subcohort along with ANGI cases diagnosed with narrow AN (F50.0) prior to 2013, and (2) ANGI cases diagnosed with narrow AN during 2013 only or with atypical AN (F50.1) by end of 2013, who were not already included in the first batch. This was done because the early narrow AN cases were genotyped along with the iPSYCH2012 sample, while the atypical and later narrow AN cases were genotyped separately. For LDpred parameters, we set the LD radius to 100 single‐nucleotide polymorphisms (SNP; which approximately equals the number of SNPs divided by 3000, the recommended LD radius for LDpred) and calculated PGS for a range of *p* parameters (expected fraction of non‐zero effects). Number of SNPs used in PGS calculations are listed in Table S1. We calculated correlations with case status of the same disorder for all tested LDpred *p* parameter thresholds (presented in Table S2) and chose the PGS that yielded the highest positive correlation for each disorder (AN, IBD: 0.1. Crohn's disease, ulcerative colitis: 0.001). Calculation of the depression PGS (*p*‐value threshold 0.2) use in our analyses has been described elsewhere (Grove et al., 2019). As we compared a limited number of models, the risk of overfitting is small. The chosen PGS were standardized separately for the two batches by subtracting the mean and dividing by the standard deviation (SD) of the subcohort.

### Statistical analysis

For the associations between diagnoses, survival analysis of the risk of being diagnosed with one disorder was conducted in the full study population using Cox proportional hazards regression by comparing individuals exposed to personal or parental history of the other disorder from date of first diagnosis to those not exposed, resulting in estimated hazard ratios (HR) with 95% confidence intervals (CI). Each person in the study population was followed from their 6^th^ birthday until the first diagnosis of the disorder of interest, death, emigration, or December 31, 2013, whichever occurred first. Analyses were adjusted for birth year quartiles, age as the underlying time scale, and sex by separate baseline hazards. Additional analyses were conducted including time since the exposure diagnoses.

The associations between PGS of one disorder and case status of the other disorder by end of 2013 was estimated in the study subpopulation using logistic regression, adjusted for sex and birth year, grouped into quartiles. Analyses including PGS were also controlled for population stratification by adjusting for the first 10 genomic principal components. The PGS were included in the logistic model as continuous variables, where results are reported as change in odds per SD. To measure the proportion of variance explained by the PGS, we report the difference in Nagelkerke's pseudo‐*R* ^2^ from the full logistic model and the null model without PGS.

To account for the case‐cohort design, individuals in the subpopulation were weighted in the logistic models according to their AN case status, with cases assigned weight equaling 1 and non‐cases assigned weight equal to the inverse of the probability of inclusion in the random subcohort, that is, the size of the eligible background population without AN diagnoses divided by the size of the random subcohort. The same approach was used in analyses for depression with differential weighting of depression cases and non‐cases.

All analyses of the association between diagnoses or between PGS and diagnoses were adjusted for parental educational level at proband age 6, defined as the highest completed education of either parent in four categories: basic school, vocational training or high school, short‐ or medium‐term higher education, and long‐term higher education.

Two‐sample MR based on summary statistics files was conducted to test for causality, and we report MR‐Egger regression results.

All analyses described above were repeated for depression, and all statistical analyses were conducted in Stata version 15 or in R version 3.6 using the TwoSampleMR package (Hemani et al., 2018). The statistical significance threshold for all results was 0.05.

## RESULTS

The 1,238,813 members of the study population were followed until a maximum age of 32 years and contributed a combined ∼17.3 million person‐years to the survival analyses. During follow‐up, 5064 individuals were diagnosed with AN, 6947 with IBD, 3461 with Crohn's disease, 4396 with ulcerative colitis, and 25,275 with depression.

Results from the survival analyses of diagnostic associations are presented in Table 1, which also lists the number of individuals exposed to the prior diagnosis at some point during follow‐up and the number of exposed cases. Results for covariates are listed in Table S3. Having a prior diagnosis of AN was associated with statistically significantly increased hazards of later diagnoses of IBD (HR: 1.44 [95% CI: 1.05–1.97]), Crohn's disease (1.60 [1.04–2.46]), and ulcerative colitis (1.66 [1.15–2.39]). Results regarding the time since the exposure diagnosis are included in Table S4. There were no statistically significant associations of a later AN diagnosis for individuals with previous diagnoses of IBD, Crohn's disease, or ulcerative colitis.

**TABLE 1:** Associations between within‐individual and within‐family diagnoses of anorexia nervosa and inflammatory bowel diseases, depending on the order of diagnoses

	Exposure diagnosis	Individuals exposed during follow‐up (*N*)	Outcome diagnosis	Exposed individuals with outcome diagnosis (*N*)	HR (95% CI)	*p*
Within‐individual diagnoses	Prior anorexia nervosa	5042	Inflammatory bowel disease	40	1.44 (1.05, 1.97)	0.024
	Prior anorexia nervosa	5055	Crohn's disease	22	1.60 (1.04, 2.46)	0.033
	Prior anorexia nervosa	5051	Ulcerative colitis	29	1.66 (1.15, 2.39)	0.007
	Prior inflammatory bowel disease	7059	Anorexia nervosa	21	1.30 (0.84, 2.02)	0.241
	Prior Crohn's disease	3521	Anorexia nervosa	9	1.04 (0.52, 2.08)	0.911
	Prior ulcerative colitis	4446	Anorexia nervosa	12	1.26 (0.71, 2.21)	0.468
Within‐family diagnoses	Parental anorexia nervosa	3072	Inflammatory bowel disease	11	0.98 (0.54, 1.77)	0.9813
	Parental anorexia nervosa	3076	Crohn's disease	5	0.87 (0.36, 2.09)	0.756
	Parental anorexia nervosa	3073	Ulcerative colitis	9	1.29 (0.70, 2.59)	0.442
	Parental inflammatory bowel disease	34,874	Anorexia nervosa	123	1.11 (0.93, 1.33)	0.262
	Parental Crohn's disease	13,222	Anorexia nervosa	46	1.11 (0.83, 1.50)	0.486
	Parental ulcerative colitis	25,433	Anorexia nervosa	90	1.12 (0.90, 1.38)	0.304

*Note*: Hazard ratios (HR) with 95% confidence intervals (CI).

We found no statistically significant results for parental diagnoses and later offspring diagnoses for any combination of diagnosis. The results for depression, presented in Table 2, were comparable to the AN results. We observed elevated rates of similar magnitude of later IBD and IBD subtypes for individuals previously diagnosed with depression, and no statistically significant association between parental and offspring diagnoses, except for increased rates of depression for individuals with at least one parent diagnosed with Crohn's disease. However, we also found increased rates of later depression in individuals previously diagnosed with IBD and IBD subtypes.

**TABLE 2:** Associations between within‐individual and within‐family diagnoses of depression and inflammatory bowel diseases, depending on the order of diagnoses

	Exposure diagnosis	Individuals exposed during follow‐up (*N*)	Outcome diagnosis	Exposed individuals with outcome diagnosis (*N*)	HR (95% CI)	*p*
Within‐individual diagnoses	Prior depression	25,091	Inflammatory bowel disease	162	1.53 (1.30, 1.79)	<0.001
	Prior depression	25,174	Crohn's disease	89	1.80 (1.46, 2.23)	<0.001
	Prior depression	25,164	Ulcerative colitis	93	1.30 (1.05, 1.62)	0.015
	Prior inflammatory bowel disease	6938	Depression	184	1.45 (1.25, 1.67)	<0.001
	Prior Crohn's disease	3454	Depression	101	1.59 (1.30, 1.93)	<0.001
	Prior ulcerative colitis	4383	Depression	111	1.39 (1.15, 1.68)	0.001
Within‐family diagnoses	Parental depression	76,013	Inflammatory bowel disease	307	0.95 (0.85, 1.07)	0.457
	Parental depression	76,093	Crohn's disease	167	1.06 (0.91, 1.24)	0.44
	Parental depression	76,072	Ulcerative colitis	190	0.92 (0.79, 1.07)	0.274
	Parental inflammatory bowel disease	34,778	Depression	602	1.02 (0.94, 1.11)	0.635
	Parental Crohn's disease	13,192	Depression	260	1.19 (1.07, 1.37)	0.006
	Parental ulcerative colitis	25,354	Depression	408	0.95 (0.86, 1.05)	0.336

*Note*: Hazard ratios (HR) with 95% confidence intervals (CI).

Associations between PGS and diagnoses of the same disorder are presented in Table S5 for validation of the PGS in the sample.

Of the study subpopulation used in the logistic analyses of association with PGS, 176 were diagnosed with IBD, 83 with Crohn's disease, and 115 with ulcerative colitis by the end of 2013. In individuals diagnosed with both AN and IBD during the follow‐up period, the median time between diagnoses was 4.5 years if AN was the prior diagnosis and 1.9 years if IBD was the prior diagnosis.

Results from the PGS analyses are presented in Table 3. We found no statistically significant patterns of association between the PGS of one disorder and case status of the other disorder in either direction, except for a negative association between ulcerative colitis PGS and AN case status (OR: 0.95 [95% CI: 0.91–0.99] per SD). In comparison, no significant associations between depression and IBD using PGS were found (Table 4).

**TABLE 3:** Associations between anorexia nervosa polygenic scores (PGS) and diagnoses of inflammatory bowel disease and subtypes, and vice versa

Exposure PGS	Outcome diagnosis	Individuals with outcome diagnosis (*N*)	OR (95% CI)/SD	*p*	Nagelkerke pseudo‐*R* ^2^		
					Null model	Full model	Difference
Anorexia nervosa	Inflammatory bowel disease	176	1.03 (0.83, 1.28)	0.767	0.794214	0.197496	0.000282
Anorexia nervosa	Crohn's disease	83	0.88 (0.66, 1.18)	0.403	0.110876	0.113495	0.002319
Anorexia nervosa	Ulcerative colitis	115	1.12 (0.84, 1.49)	0.446	0.189134	0.191425	0.002291
Inflammatory bowel disease	Anorexia nervosa	4271	1.04 (0.99, 1.08)	0.115	0.231002	0.231205	0.000203
Crohn's disease	Anorexia nervosa	4271	1.03 (0.99, 1.08)	0.150	0.231002	0.231457	0.000456
Ulcerative colitis	Anorexia nervosa	4271	0.95 (0.91, 0.99)	0.020	0.231002	0.231163	0.000161

*Note*: Odds ratios (OR) with 95% confidence intervals (CI) per one standard deviation (SD) increase in PGS.

**TABLE 4:** Associations between depression polygenic score (PGS) and diagnoses of inflammatory bowel disease and its subtypes, and vice versa

Exposure PGS	Outcome diagnosis	Individuals with outcome diagnosis (*N*)	OR (95% CI)/SD	*p*	Nagelkerke pseudo‐*R* ^2^		
					Null model	Full model	Difference
Depression	Inflammatory bowel disease	396	0.99 (0.80, 1.22)	0.914	0.130244	0.130263	0.000019
Depression	Crohn's disease	199	0.92 (0.70, 1.22)	0.568	0.108187	0.108911	0.000724
Depression	Ulcerative colitis	244	0.95 (0.72, 1.25)	0.725	0.106734	0.107045	0.000031
Inflammatory bowel disease	Depression	18,761	1.00 (0.98, 1.03)	0.803	0.328959	0.328963	0.000004
Crohn's disease	Depression	18,761	1.00 (0.98, 1.03)	0.779	0.328959	0.328964	0.000005
Ulcerative colitis	Depression	18,761	0.98 (0.95, 1.01)	0.219	0.328959	0.329054	0.000095

*Note*: Odds ratios (OR) with 95% confidence intervals (CI) per one standard deviation (SD) increase in PGS.

None of the MR analyses yielded significant MR‐Egger slope estimates, shown in Table S6, indicating no causal genetic relationship between AN and IBD in either direction. We also found no evidence for a causal relationship between depression and IBD.

## DISCUSSION

In this study of diagnostic and genetic associations between AN and IBD, we found that having a prior diagnosis of AN was associated with increased hazard of later being diagnosed with IBD including specifically Crohn's disease and ulcerative colitis. We found no evidence of association between prior IBD and later AN, between parental and offspring diagnoses, or between PGS of one disorder and onset of the other. Based on the currently available data, we found no indication of causal genetic relationships between the two conditions. Any associations we did find were replicated for associations between IBD and depression as well.

The positive associations between prior AN and later onset of IBD could in part point to a non‐genetic relationship between the disorders. AN behaviors including persistent restrictive food intake, self‐induced vomiting, and laxative misuse may negatively impact the function of the digestive tract and result in persistent gastrointestinal complications (Salvioli et al., 2013; Zipfel et al., 2006). Clinical studies have found that individuals suffering from AN have atypical intestinal microbiota and lower microbial diversity (Armougom et al., 2009; Avila et al., 2019; Bulik et al., 2019; Glenny et al., 2017; Pfleiderer et al., 2013; Roubalova et al., 2020; Seitz et al., 2019), and it has been proposed that IBD are associated with microbiota dysbiosis and inappropriate immune response to the gut microbes, which aggravate gut inflammation (Tamboli et al., 2004; Zuo & Ng, 2018). Given the inflammatory component to IBD and the established associations between infections, autoinflammatory illnesses and eating disorders (Breithaupt et al., 2019; Zerwas et al., 2017), any association could be due to shared inflammatory and immunological pathophysiology. It is also possible that the associations between prior AN and later IBD could be explained by shared environmental risk factors not accounted for in our analyses. Although we did not find significant associations in either direction, it is possible that a longer follow‐up period and the addition of more individuals to the cohort could change these results, and as the robustness of the AN PGS will improve with increasing AN GWAS sample size.

The fact that we found comparable associations between prior depression and later IBD show that the relationship is not specific to AN among psychiatric disorders, although the exact pathways for these associations may differ. For example, depression is associated with increased cortisol levels and reduced sensitivity of glucocorticoid receptors, which can enhance inflammatory responses (Frolkis et al., 2019) and increase the risk of IBD.

We did not observe any statistically significant association between prior IBD and later AN. IBD can occur at any age, but peak age of onset is between ages 20 and 40 (Malik, 2015), and with a young cohort with ages ranging between 8 and 32 years and not having passed through the peak age of onset period, few individuals have been exposed to first IBD and later AN. Older longitudinal cohorts may yield different results. That a potential association could exist seems reasonable, as the pain and discomfort often associated with eating for IBD patients could lead to increased focus on diet, avoiding certain foods, altered eating behavior, and weight loss, similar to what is seen in disordered eating (Conviser et al., 2018). In a population‐based Swedish study of associations between autoimmune illnesses and eating disorders, Hedman et al. reported statistically significant bidirectional associations between AN and Crohn's disease in women (Hedman et al., 2019). Furthermore, Zerwas et al. reported a statistically significant association between prior gastrointestinal disorders (including Crohn's disease and ulcerative colitis) and later AN in a study based on the entire Danish population (Zerwas et al., 2017). As IBD was included among a number of autoinflammatory diseases with gastrointestinal involvement, it is not possible to directly compare the results to ours, despite the overlap in samples. In contrast to our AN findings, we found statistically significant associations between prior IBD and later depression. Inflammation as risk factor for depression is well‐established (Köhler et al., 2016), and as depression generally has a later onset than AN, our study design using the present data was better suited to detect associations of prior IBD and later depression.

### Strengths and limitations

We used a population‐based main study sample and a case‐cohort subcohort, including one that was chosen at random from the Danish population, and blood samples collected shortly after birth from nearly all individuals born in Denmark were obtained from a national biobank. This approach minimizes biases affecting selection into the study sample. The young age of the study population could explain some of the non‐significant results, especially regarding prior diagnoses of IBD, and we expect that reliability will improve with longer follow‐up.

We found no evidence of causality due to shared genetics between AN and IBD. However, the summary statistics files used in the MR analyses were based on relatively small samples. In particular, only eight genome‐wide significant SNPs have been identified for AN, and PGS for IBD, Crohn's disease and ulcerative colitis were not significantly associated with diagnoses of the same disease in our sample (see Table S3), limiting the confidence of any conclusions.

History of diagnoses was obtained from national health registers, which eliminates recall bias. However, Danish health registers only include diagnoses given in hospital and not in primary care. This could mean that we only capture the most severe cases of both AN, IBD and depression. We were unable to adjust for any other comorbidities that may play a role in the association between AN and IBD due to the relatively low number of comorbid cases. It is not possible to discern to what degree misdiagnosis could explain the association seen in this study. Eating disorders and IBD share common features and symptoms, such as weight loss and altered eating behavior, and misdiagnosis between the two have been reported in the literature (Gryboski, 1993; Hershman & Hershman, 1985; Jenkins et al., 1988). Although misdiagnosing AN as an IBD is possible, IBD diagnoses in the Danish health registers have high validity (Fonager et al., 1996), with positive predictive values for diagnoses given in Danish hospitals of 97% for Crohn's disease and 90% for ulcerative colitis. Although the validity of IBD diagnoses in Danish registers have been found to increase with the requirement of at least two separate diagnoses (Lophaven et al., 2017), we were unable to conduct the analyses using this approach due to low counts of exposed cases. Although register‐based diagnoses of AN have not yet been validated in Denmark, the likelihood of IBD being misdiagnosed as AN is low, given that an AN diagnosis requires not only weight loss sustained through food restriction and compensatory measures, but also an intense fear of weight gain and a self‐perception of being too fat, which is unique to AN.

## CONCLUSION

Our results point to a relationship between diagnoses of AN and IBD. We found no evidence of genetic or causal explanations, although further studies into this field may identify possible mechanisms and help identify individuals at greater risk of both AN and IBD.

Comorbidity of AN and IBD can potentially complicate treatment of either illness. Clinicians treating patients with AN should be aware of these associations and that IBD is a potential differential diagnosis or may present as a later emergent disease. When treating AN, clinicians may also consider whether gastrointestinal symptoms are not just sequelae of AN, but could in fact be manifestations of IBD.

## CONFLICT OF INTERESTS

Cynthia M. Bulik is a grant recipient and Scientific Advisory Board member of Shire, a consultant for Idorsia, and author and royalty recipient from Pearson. She is also a member of the Editorial Advisory Board for JCPP *Advances*. The remaining authors have declared that they have no competing or potential conflicts of interest. [Corrections made on 22 June 2022, after first online publication: This Conflict of Interests statement has been updated in this version.]

## AUTHOR CONTRIBUTIONS

Janne Tidselbak Larsen, Cynthia M. Bulik, Laura M. Thornton, and Liselotte Vogdrup Petersen conceived the research question and design. Funding acquisition and data collection was done by Thomas Werge, David M. Hougaard, Preben Bo Mortensen, and Cynthia M. Bulik. Data management and analysis was done by Janne Tidselbak Larsen, supervised by Liselotte Vogdrup Petersen, Zeynep Yilmaz, and Bjarni Jóhann Vilhjálmsson. Interpretation of results was done by Janne Tidselbak Larsen, Zeynep Yilmaz, Bjarni Jóhann Vilhjálmsson, Laura M. Thornton, Michael Eriksen Benros, Katherine L. Musliner, Cynthia M. Bulik, and Liselotte Vogdrup Petersen. First draft was written by Janne Tidselbak Larsen. All authors reviewed and edited drafts and approved the final draft.

## ETHICS STATEMENT

The study was approved by the Danish Data Protection Agency (AU DT ID: 2015‐57‐0002 – AU LBNR: 565; AU DT ID: 2015‐57‐098 – AU LBNR: 760), the Danish Health Data Authority (SDS FSE ID: 98; 672; 1722; 1999), and the Danish National Committee on Health Research Ethics (ID: 1‐10‐72‐287‐12).

## Supporting information

Supporting Information S1

## Data Availability

Research data are not shared.
