# Supplementary material for: Anorexia nervosa and inflammatory bowel diseases—Diagnostic and genetic associations
Source: JCPP Adv. 2021 Sep 27;1(4):e12036. doi: 10.1002/jcv2.12036 (PMC10242845; doi:10.1002/jcv2.12036)
Supplement: Supplementary file 1 — Supporting Information S1 [file JCV2-1-e12036-s001.docx]

**Eating Disorders Working Group of the Psychiatric Genomics Consortium**

(Lophaven, Lynge, & Burisch, 2017)

Roger AH Adan ^1, 2, 3^

Lars Alfredsson ^4^

Tetsuya Ando ^5^

Ole A Andreassen ^6^

Harald Aschauer ^7^

Jessica H Baker ^8^

Vladimir Bencko ^9^

Andrew W Bergen ^10, 11^

Wade H Berrettini ^12^

Andreas Birgegård ^13, 14, 15^

Joseph M Boden ^16^

Ilka Boehm ^17^

Claudette Boni ^18^

Vesna Boraska Perica ^19, 20^

Harry Brandt ^21^

Gerome Breen ^22, 23^

Julien Bryois ^15^

Katharina Buehren ^24^

Cynthia M Bulik ^8, 15, 25^

Roland Burghardt ^26^

Laura Carlberg ^27^

Matteo Cassina ^28^

Sven Cichon ^29, 30, 31^

Maurizio Clementi ^28^

Jonathan RI Coleman ^22, 23^

Roger D Cone ^32^

Philippe Courtet ^33^

Steven Crawford ^21^

Scott Crow ^34^

James J Crowley ^13, 35^

Unna N Danner ^2^

Oliver SP Davis ^36, 37^

Martina de Zwaan ^38^

George Dedoussis ^39^

Daniela Degortes ^40^

Janiece E DeSocio ^41^

Danielle M Dick ^42, 43, 44^

Dimitris Dikeos ^45^

Christian Dina ^46^

Monika Dmitrzak-Weglarz ^47^

Elisa Docampo Martinez ^48, 49, 50^

Laramie E Duncan ^51^

Karin Egberts ^52^

Stefan Ehrlich ^17^

Geòrgia Escaramís ^48, 49, 50^

Tõnu Esko ^53, 54^

Thomas Espeseth ^55^

Xavier Estivill ^48, 49, 50, 56^

Anne Farmer ^22^

Angela Favaro ^40^

Fernando Fernández-Aranda ^57, 58^

Manfred M Fichter ^59, 60^

Krista Fischer ^53^

James AB Floyd ^61^

Manuel Föcker ^62^

Lenka Foretova ^63^

Andreas J Forstner ^30, 64, 65, 66^

Monica Forzan ^28^

Christopher S Franklin ^19^

Steven Gallinger ^67^

Giovanni Gambaro ^68^

Héléna A Gaspar ^22, 23^

Ina Giegling ^69^

Johanna Giuranna ^70^

Paola Giusti-Rodríquez ^35^

Fragiskos Gonidakis ^71^

Scott Gordon ^72^

Philip Gorwood ^73, 74^

Monica Gratacos Mayora ^48, 49, 50^

Jakob Grove ^75, 76, 77, 78^

Sébastien Guillaume ^33^

Yiran Guo ^79^

Hakon Hakonarson ^79, 80^

Katherine A Halmi ^81^

Ken B Hanscombe ^82^

Konstantinos Hatzikotoulas ^19, 83^

Joanna Hauser ^84^

Johannes Hebebrand ^70^

Sietske G Helder ^22, 85^

Anjali K Henders ^86^

Stefan Herms ^29, 30^

Beate Herpertz-Dahlmann ^24^

Wolfgang Herzog ^87^

Anke Hinney ^70^

L. John Horwood ^16^

Christopher Hübel ^15, 22^

Laura M Huckins ^88^

James I Hudson ^89^

Hartmut Imgart ^90^

Hidetoshi Inoko ^91^

Vladimir Janout ^92^

Susana Jiménez-Murcia ^57, 58^

Craig Johnson ^93^

Jennifer Jordan ^94, 95^

Antonio Julià ^96^

Gursharan Kalsi ^22^

Deborah Kaminská ^97^

Allan S Kaplan ^98, 99, 100^

Jaakko Kaprio ^101, 102^

Leila Karhunen ^103^

Andreas Karwautz ^104^

Martien JH Kas ^1, 105^

Walter H Kaye ^106^

James L Kennedy ^98, 99, 100^

Martin A Kennedy ^107^

Anna Keski-Rahkonen ^101^

Kirsty Kiezebrink ^108^

Youl-Ri Kim ^109^

Katherine M Kirk ^72^

Lars Klareskog ^110^

Kelly L Klump ^111^

Gun Peggy S Knudsen ^112^

Maria C La Via ^8^

Mikael Landén ^15, 113^

Janne T Larsen ^76, 114, 115^

Stephanie Le Hellard ^116, 117, 118^

Virpi M Leppä ^15^

Robert D Levitan ^99^

Dong Li ^79^

Paul Lichtenstein ^15^

Lisa Lilenfeld ^119^

Bochao Danae Lin ^1^

Jolanta Lissowska ^120^

Astri Lundervold ^121^

Jurjen Luykx ^1^

Pierre J Magistretti ^122,123^

Mario Maj ^124^

Katrin Mannik ^53, 125^

Sara Marsal ^96^

Christian R Marshall ^126^

Nicholas G Martin ^72^

Manuel Mattheisen ^13, 14, 75, 127^

Morten Mattingsdal ^6^

Sara McDevitt ^128, 129^

Peter McGuffin ^22^

Sarah E Medland ^72^

Andres Metspalu ^53, 130^

Ingrid Meulenbelt ^131^

Nadia Micali ^132, 133^

James Mitchell ^134^

Karen Mitchell ^135, 136^

Palmiero Monteleone ^137^

Alessio Maria Monteleone ^124^

Grant W Montgomery ^72, 86, 138^

Preben Bo Mortensen ^76, 114, 115^

Melissa A Munn-Chernoff ^8^

Benedetta Nacmias ^139^

Marie Navratilova ^63^

Ioanna Ntalla ^39^

Catherine M Olsen ^140^

Roel A Ophoff ^141, 142^

Julie K O'Toole ^143^

Leonid Padyukov ^110^

Aarno Palotie ^54, 102, 144^

Jacques Pantel ^18^

Hana Papezova ^97^

Richard Parker ^72^

John F Pearson ^145^

Nancy L Pedersen ^15^

Liselotte V Petersen ^76, 114, 115^

Dalila Pinto ^88^

Kirstin L Purves ^22^

Anu Raevuori ^101^

Nicolas Ramoz ^18^

Ted Reichborn-Kjennerud ^112, 146^

Valdo Ricca ^147^

Samuli Ripatti ^148^

Stephan Ripke ^149, 150, 151^

Franziska Ritschel ^17, 152^

Marion Roberts ^22^

Alessandro Rotondo ^153^

Dan Rujescu ^69^

Filip Rybakowski ^154^

Paolo Santonastaso ^155^

André Scherag ^156^

Stephen W Scherer ^157, 158^

Ulrike Schmidt ^22^

Nicholas J Schork ^159^

Alexandra Schosser ^160^

Jochen Seitz ^24^

Lenka Slachtova ^161^

P. Eline Slagboom ^131^

Margarita CT Slof-Op 't Landt ^162, 163^

Agnieszka Slopien ^164^

Nicole Soranzo ^19, 165, 166, 167^

Sandro Sorbi ^139, 168^

Lorraine Southam ^19^

Vidar W Steen ^169, 170^

Michael Strober ^171, 172^

Garret D Stuber ^8, 173^

Patrick F Sullivan ^8, 15, 35^

Beata Świątkowska ^174^

Jin P Szatkiewicz ^35^

Ioanna Tachmazidou ^19^

Elena Tenconi ^40^

Laura M Thornton ^8^

Alfonso Tortorella ^175, 176^

Federica Tozzi ^177^

Janet Treasure ^22^

Artemis Tsitsika ^178^

Marta Tyszkiewicz-Nwafor ^164^

Konstantinos Tziouvas ^179^

Annemarie A van Elburg ^2, 180^

Eric F van Furth ^162, 163^

Tracey D Wade ^181^

Gudrun Wagner ^104^

Esther Walton ^17^

Hunna J Watson ^8, 182, 183^

Thomas Werge ^184^

David C Whiteman ^140^

H.-Erich Wichmann ^185^

Elisabeth Widen ^102^

D. Blake Woodside ^99, 100, 186, 187^

Shuyang Yao ^15^

Zeynep Yilmaz ^8, 35^

Eleftheria Zeggini ^19, 83^

Stephanie Zerwas ^8^

Stephan Zipfel ^188^

1. Brain Center Rudolf Magnus, Department of Translational Neuroscience, University Medical Center Utrecht, Utrecht, The Netherlands
2. Center for Eating Disorders Rintveld, Altrecht Mental Health Institute, Zeist, The Netherlands
3. Sahlgrenska Academy, University of Gothenburg, Gothenburg, Sweden
4. Institute of Environmental Medicine, Karolinska Institutet, Stockholm, Sweden
5. Department of Behavioral Medicine, National Institute of Mental Health, National Center of Neurology and Psychiatry, Kodaira, Tokyo, Japan
6. NORMENT Centre, Division of Mental Health and Addiction, University of Oslo, Oslo University Hospital, Oslo, Norway
7. Biopsychosocial Corporation, Vienna, Austria
8. Department of Psychiatry, University of North Carolina at Chapel Hill, Chapel Hill, North Carolina, US
9. First Faculty of Medicine, Institute of Hygiene and Epidemiology, Charles University, Prague, Czech Republic
10. BioRealm, LLC, Walnut, California, USA
11. Oregon Research Institute, Eugene, Oregon, USA
12. Department of Psychiatry, Center for Neurobiology and Behavior, University of Pennsylvania Perelman School of Medicine, Philadelphia, Pennsylvania, USA
13. Department of Clinical Neuroscience, Karolinska Institutet, Stockholm, Sweden
14. Center for Psychiatry Research, Stockholm Health Care Services, Stockholm City Council, Stockholm, Sweden
15. Department of Medical Epidemiology and Biostatistics, Karolinska Institutet, Stockholm, Sweden
16. Christchurch Health and Development Study, University of Otago, Christchurch, New Zealand
17. Division of Psychological and Social Medicine and Developmental Neurosciences, Faculty of Medicine, Technische Universität Dresden, Dresden, Germany
18. INSERM U894, Centre of Psychiatry and Neuroscience, Paris, France
19. Wellcome Sanger Institute, Wellcome Genome Campus, Hinxton, Cambridge, UK
20. Department of Medical Biology, School of Medicine, University of Split, Split, Croatia
21. The Center for Eating Disorders at Sheppard Pratt, Baltimore, Maryland, US
22. Institute of Psychiatry, Psychology and Neuroscience, Social, Genetic and Developmental Psychiatry (SGDP) Centre, King’s College London, London, UK
23. National Institute for Health Research Biomedical Research Centre, King’s College London and South London and Maudsley National Health Service Trust, London, UK
24. Department of Child and Adolescent Psychiatry, Psychosomatics and Psychotherapy, RWTH Aachen University, Aachen, Germany
25. Department of Nutrition, University of North Carolina at Chapel Hill, Chapel Hill, North Carolina, US
26. Klinikum Frankfurt/Oder, Frankfurt, Germany
27. Medical University of Vienna, Vienna, Austria
28. Clinical Genetics Unit, Department of Woman and Child Health, University of Padova, Padova, Italy
29. Institute of Medical Genetics and Pathology, University Hospital Basel, Basel, Switzerland
30. Department of Biomedicine, University of Basel, Basel, Switzerland
31. Institute of Neuroscience and Medicine (INM-1), Research Center Juelich, Juelich, Germany
32. Life Sciences Institute and Department of Molecular and Integrative Physiology, University of Michigan, Ann Arbor, Michigan, US
33. Department of Emergency Psychiatry and Post-Acute Care, CHRU Montpellier, University of Montpellier, Montpellier, France
34. Department of Psychiatry, University of Minnesota, Minneapolis, Minnesota, US
35. Department of Genetics, University of North Carolina at Chapel Hill, Chapel Hill, North Carolina, US
36. MRC Integrative Epidemiology Unit, University of Bristol, Bristol, UK
37. School of Social and Community Medicine, University of Bristol, Bristol, UK
38. Department of Psychosomatic Medicine and Psychotherapy, Hannover Medical School, Hannover, Germany
39. Department of Nutrition and Dietetics, Harokopio University, Athens, Greece
40. Department of Neurosciences, University of Padova, Padova, Italy
41. College of Nursing, Seattle University, Seattle, Washington, US
42. Department of Psychology, Virginia Commonwealth University, Richmond, Virginia, US
43. College Behavioral and Emotional Health Institute, Virginia Commonwealth University, Richmond, Virginia, US
44. Department of Human & Molecular Genetics, Virginia Commonwealth University, Richmond, Virginia, US
45. Department of Psychiatry, Athens University Medical School, Athens University, Athens, Greece
46. L'institut du thorax, INSERM, CNRS, UNIV Nantes, Nantes, France
47. Department of Psychiatric Genetics, Poznan University of Medical Sciences, Poznan, Poland
48. Barcelona Institute of Science and Technology, Barcelona, Spain
49. Universitat Pompeu Fabra, Barcelona, Spain
50. Centro de Investigación Biomédica en Red en Epidemiología y Salud Pública (CIBERESP), Barcelona, Spain
51. Department of Psychiatry and Behavioral Sciences, Stanford University, Stanford, California, US
52. Department of Child and Adolescent Psychiatry, Psychosomatics and Psychotherapy, University Hospital of Würzburg, Centre for Mental Health, Würzburg, Germany
53. Estonian Genome Center, University of Tartu, Tartu, Estonia
54. Program in Medical and Population Genetics, Broad Institute of the Massachusetts Institute of Technology and Harvard University, Cambridge, Massachusetts, US
55. Department of Psychology, University of Oslo, Oslo University Hospital, Oslo, Norway
56. Genomics and Disease, Bioinformatics and Genomics Programme, Centre for Genomic Regulation, Barcelona, Spain
57. Department of Psychiatry, University Hospital of Bellvitge –IDIBELL and CIBERobn, Barcelona, Spain
58. Department of Clinical Sciences, School of Medicine, University of Barcelona, Barcelona, Spain
59. Department of Psychiatry and Psychotherapy, Ludwig‐Maximilians‐University (LMU), Munich, Germany
60. Schön Klinik Roseneck affiliated with the Medical Faculty of the University of Munich, Munich,Germany
61. Genomics PLC, Oxford, UK
62. Department of Child and Adolescent Psychiatry, University of Münster, Münster, Germany
63. Department of Cancer, Epidemiology and Genetics, Masaryk Memorial Cancer Institute, Brno, Czech Republic
64. Centre for Human Genetics, University of Marburg, Marburg, Germany
65. Institute of Human Genetics, University of Bonn, School of Medicine & University Hospital Bonn, Bonn, Germany
66. Department of Psychiatry (UPK), University of Basel, Basel, Switzerland
67. Department of Surgery, Faculty of Medicine, University of Toronto, Toronto, Ontario, Canada
68. Division of Nephrology and Dialysis, Institute of Internal Medicine and Medical Specialties, Columbus-Gemelli University Hospital, Rome, Italy
69. Department of Psychiatry, Psychotherapy and Psychosomatics, Martin Luther University of Halle-Wittenberg, Halle (Saale), Germany
70. Department of Child and Adolescent Psychiatry, University Hospital Essen, University of Duisburg-Essen, Essen, Germany
71. 1st Psychiatric Department, National and Kapodistrian University of Athens, Medical School, Eginition Hospital, Athens, Greece
72. QIMR Berghofer Medical Research Institute, Brisbane, Queensland, Australia
73. INSERM U1266, Institute of Psychiatry and Neuroscience of Paris, Paris, France
74. CMME (GHU Paris Psychiatrie et Neurosciences), Paris Descartes University, Paris, France
75. Department of Biomedicine, Aarhus University, Aarhus, Denmark
76. The Lundbeck Foundation Initiative for Integrative Psychiatric Research (iPSYCH), Aarhus, Denmark
77. Centre for Integrative Sequencing, iSEQ, Aarhus University, Aarhus, Denmark
78. Bioinformatics Research Centre, Aarhus University, Aarhus, Denmark
79. Center for Applied Genomics, Children's Hospital of Philadelphia, Philadelphia, Pennsylvania, US
80. Department of Pediatrics, University of Pennsylvania Perelman School of Medicine, Philadelphia, Pennsylvania, US
81. Department of Psychiatry, Weill Cornell Medical College, New York, New York, US
82. Department of Medical and Molecular Genetics, King's College London, Guy’s Hospital, London, UK
83. Institute of Translational Genomics, Helmholtz Zentrum München - German Research Centre for Environmental Health, Neuherberg, Germany
84. Department of Adult Psychiatry, Poznan University of Medical Sciences, Poznan, Poland
85. Zorg op Orde, Delft, The Netherlands
86. Institute for Molecular Bioscience, University of Queensland, Brisbane, Queensland, Australia
87. Department of General Internal Medicine and Psychosomatics, Heidelberg University Hospital, Heidelberg University, Heidelberg, Germany
88. Department of Psychiatry, and Genetics and Genomics Sciences Division of Psychiatric Genomics, Icahn School of Medicine at Mount Sinai, New York, New York, US
89. Biological Psychiatry Laboratory, McLean Hospital/Harvard Medical School, Boston, Massachusetts, US
90. Eating Disorders Unit, Parklandklinik, Bad Wildungen, Germany
91. Department of Molecular Life Science Division of Basic Medical Science and Molecular Medicine, School of Medicine, Tokai University, Isehara, Japan
92. Faculty of Health Sciences, Palacky University, Olomouc, Czech Republic
93. Eating Recovery Center, Denver, Colorado, US
94. Department of Psychological Medicine, University of Otago, Christchurch, New Zealand
95. Canterbury District Health Board, Christchurch, New Zealand
96. Rheumatology Research Group, Vall d’Hebron Research Institute, Barcelona, Spain
97. Department of Psychiatry, First Faculty of Medicine, Charles University, Prague, Czech Republic
98. Centre for Addiction and Mental Health, Toronto, Ontario, Canada
99. Institute of Medical Science, University of Toronto, Toronto, Ontario, Canada
100. Department of Psychiatry, University of Toronto, Toronto, Ontario, Canada
101. Department of Public Health, University of Helsinki, Helsinki, Finland
102. Institute for Molecular Medicine FIMM, HiLIFE, University of Helsinki, Helsinki, Finland
103. Institute of Public Health and Clinical Nutrition, Department of Clinical Nutrition, University of Eastern Finland, Kuopio, Finland
104. Eating Disorders Unit, Department of Child and Adolescent Psychiatry, Medical University of Vienna, Vienna, Austria
105. Groningen Institute for Evolutionary Life Sciences, University of Groningen, Groningen, The Netherlands
106. Department of Psychiatry, University of California San Diego, San Diego, California, US
107. Department of Pathology and Biomedical Science, University of Otago, Christchurch, New Zealand
108. Institute of Applied Health Sciences, School of Medicine, Medical Sciences and Nutrition, University of Aberdeen, Aberdeen, UK
109. Department of Psychiatry, Seoul Paik Hospital, Inje University, Seoul, Korea
110. Rheumatology Unit, Department of Medicine, Center for Molecular Medicine, Karolinska Institutet and Karolinska University Hospital, Stockholm, Sweden
111. Department of Psychology, Michigan State University, East Lansing, Michigan, US
112. Department of Mental Disorders, Norwegian Institute of Public Health, Oslo, Norway
113. Department of Psychiatry and Neurochemistry Institute of Neuroscience and Physiology, The Sahlgrenska Academy at the University of Gothenburg, Gothenburg, Sweden
114. National Centre for Register-Based Research, Aarhus BSS, Aarhus University, Aarhus, Denmark
115. Centre for Integrated Register-based Research (CIRRAU), Aarhus University, Aarhus, Denmark
116. Department of Clinical Science, K.G. Jebsen Centre for Psychosis Research, Norwegian Centre for Mental Disorders Research (NORMENT), University of Bergen, Bergen, Norway
117. Dr. Einar Martens Research Group for Biological Psychiatry, Center for Medical Genetics and Molecular Medicine, Haukeland University Hospital, Bergen, Norway
118. Department of Clinical Medicine, Laboratory Building, Haukeland University Hospital, Bergen, Norway
119. The Chicago School of Professional Psychology, Washington DC, US
120. Department of Cancer Epidemiology and Prevention, M Skłodowska-Curie Cancer Center - Oncology Center, Warsaw, Poland
121. University of Bergen, K. G. Jebsen Center for Neuropsychiatric Disorders, Bergen, Norway
122. BESE Division, King Abdullah University of Science and Technology, Thuwal, Saudi Arabia
123. Department of Psychiatry, University of Lausanne-University Hospital of Lausanne (UNIL-CHUV), Lausanne, Switzerland
124. Department of Psychiatry, University of Campania "Luigi Vanvitelli", Naples, Italy
125. Center for Integrative Genomics, University of Lausanne, Lausanne, Switzerland
126. Department of Paediatric Laboratory Medicine Division of Genome Diagnostics, The Hospital for Sick Children, Toronto, Ontario, Canada
127. Department of Psychiatry, Psychosomatics and Psychotherapy, University of Würzburg, Würzburg, Germany
128. Department of Psychiatry, University College Cork, Cork, Ireland
129. Eist Linn Adolescent Unit, Bessborough, Health Service Executive South, Cork, Ireland
130. Institute of Molecular and Cell Biology, University of Tartu, Tartu, Estonia
131. Molecular Epidemiology Section (Department of Biomedical Datasciences), Leiden University Medical Centre, Leiden, The Netherlands
132. Department of Psychiatry, Faculty of Medicine, University of Geneva, Geneva, Switzerland
133. Division of Child and Adolescent Psychiatry, Geneva University Hospital, Geneva, Switzerland
134. Department of Psychiatry and Behavioral Science, University of North Dakota School of Medicine and Health Sciences, Fargo, North Dakota, US
135. National Center for PTSD, VA Boston Healthcare System, Boston, Massachusetts, US
136. Department of Psychiatry, Boston University School of Medicine, Boston, Massachusetts, US
137. Department of Medicine, Surgery and Dentistry "Scuola Medica Salernitana", University of Salerno, Salerno, Italy
138. Queensland Brain Institute, University of Queensland, Brisbane, Queensland, Australia
139. Department of Neuroscience, Psychology, Drug Research and Child Health (NEUROFARBA), University of Florence, Florence, Italy
140. Population Health Department, QIMR Berghofer Medical Research Institute, Brisbane, Queensland, Australia
141. Center for Neurobehavioral Genetics Semel Institute for Neuroscience and Human Behavior, University of California Los Angeles, Los Angeles, California, USA
142. Department of Psychiatry, Erasmus MC, University Medical Center Rotterdam, Rotterdam, The Netherlands
143. Kartini Clinic, Portland, Oregon, US
144. Center for Human Genome Research, Massachusetts General Hospital, Boston, Massachusetts, USA
145. Biostatistics and Computational Biology Unit,  University of Otago, Christchurch, New Zealand
146. Institute of Clinical Medicine, University of Oslo, Oslo, Norway
147. Department of Health Science, University of Florence, Florence, Italy
148. Department of Biometry, University of Helsinki, Helsinki, Finland
149. Analytic and Translational Genetics Unit, Department of Medicine, Massachusetts General Hospital and Harvard Medical School, Boston, Massachusetts, US
150. Stanley Center for Psychiatric Research, Broad Institute of the Massachusetts Institute of Technology and Harvard University, Cambridge, Massachusetts, US
151. Department of Psychiatry and Psychotherapy, Charité - Universitätsmedizin, Berlin, Germany
152. Eating Disorders Research and Treatment Center, Department of Child and Adolescent Psychiatry, Faculty of Medicine, Technische Universität Dresden, Dresden, Germany
153. Department of Psychiatry, Neurobiology, Pharmacology, and Biotechnologies, University of Pisa, Pisa, Italy
154. Department of Psychiatry, Poznan University of Medical Sciences, Poznan, Poland
155. Department of Neurosciences, Padua Neuroscience Center, University of Padova, Padova, Italy
156. Institute of Medical Statistics, Computer and Data Sciences, Jena University Hospital, Jena, Germany
157. Department of Genetics and Genomic Biology, The Hospital for Sick Children, Toronto, Ontario, Canada
158. McLaughlin Centre, University of Toronto, Toronto, Ontario, Canada
159. J. Craig Venter Institute (JCVI), La Jolla, California, US
160. Department of Psychiatry and Psychotherapy, Medical University of Vienna, Vienna, Austria
161. Department of Pediatrics and Center of Applied Genomics, First Faculty of Medicine, Charles University, Prague, Czech Republic
162. Center for Eating Disorders Ursula, Rivierduinen, Leiden, The Netherlands
163. Department of Psychiatry, Leiden University Medical Centre, Leiden, The Netherlands
164. Department of Child and Adolescent Psychiatry, Poznan University of Medical Sciences, Poznan, Poland
165. Donor Health and Genomics, National Institute for Health Research Blood and Transplant Unit, Cambridge, UK
166. Division of Cardiovascular Medicine, British Heart Foundation Centre of Excellence, Cambridge, UK
167. Department of Haematology, University of Cambridge, Cambridge, UK
168. IRCCS Fondazione Don Carlo Gnocchi, Florence, Italy
169. Center for Medical Genetics and Molecular Medicine, Haukeland University Hospital, Bergen, Norway
170. Department of Clinical Science, University of Bergen, Bergen, Norway
171. Department of Psychiatry and Biobehavioral Science, Semel Institute for Neuroscience and Human Behavior, University of California Los Angeles, Los Angeles, California, US
172. David Geffen School of Medicine, University of California Los Angeles, Los Angeles, California, US
173. Department of Cell Biology and Physiology, University of North Carolina at Chapel Hill, Chapel Hill, North Carolina, US
174. Department of Environmental Epidemiology, Nofer Institute of Occupational Medicine, Lodz, Poland
175. Department of Psychiatry, University of Naples SUN, Naples, Italy
176. Department of Psychiatry, University of Perugia, Perugia, Italy
177. Brain Sciences Department, Stremble Ventures, Limassol, Cyprus
178. Adolescent Health Unit, Second Department of Pediatrics, "P. & A. Kyriakou" Children's Hospital, University of Athens, Athens, Greece
179. Pediatric Intensive Care Unit, "P. & A. Kyriakou" Children's Hospital, University of Athens, Athens, Greece
180. Faculty of Social and Behavioral Sciences, Utrecht University, Utrecht, The Netherlands
181. School of Psychology, Flinders University, Adelaide, South Australia, Australia
182. School of Psychology, Curtin University, Perth, Western Australia, Australia
183. School of Paediatrics and Child Health, University of Western Australia, Perth, Western Australia, Australia
184. Department of Clinical Medicine, University of Copenhagen, Copenhagen, Denmark
185. Helmholtz Centre Munich - German Research Center for Environmental Health, Munich, Germany
186. Centre for Mental Health, University Health Network, Toronto, Ontario, Canada
187. Program for Eating Disorders, University Health Network, Toronto, Ontario, Canada
188. Department of Internal Medicine VI, Psychosomatic Medicine and Psychotherapy, University Medical Hospital Tuebingen, Tuebingen, Germany

Figure S1. Flowchart depicting the inclusion of individuals in the study population and in the study subpopulation for the comparison disorder depression.

*
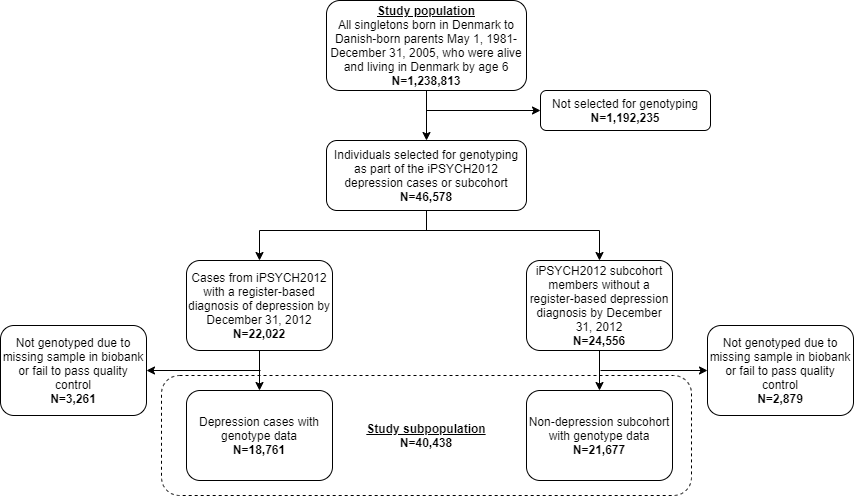
*

Table S1. Number of single-nucleotide polymorphisms (SNPs) used in polygenic score calculations for each disorder.

|  | **Number of SNPs** | |
| --- | --- | --- |
|  | **Batch 1^1^** | **Batch 2^2^** |
| Anorexia nervosa | 211,622 | 210,274 |
| Inflammatory bowel disease | 217,685 | 215,425 |
| Crohn's disease | 217,610 | 215,353 |
| Ulcerative colitis | 217,622 | 215,361 |

^1^Batch 1: iPSYCH subcohort along with Anorexia Nervosa Genetics Initiative (ANGI) cases diagnosed with narrow anorexia nervosa (F50.0) prior to 2013

^2^Batch 2: ANGI cases diagnosed with narrow anorexia nervosa during 2013 only or with atypical anorexia nervosa (F50.1) by end of 2013, who were not already included in Batch 1

Table S2. Pearson’s correlation coefficients between standardized polygenic scores and diagnoses of the same disorder, presented for each disorder and each LDpred p threshold.

|  | **Pearson’s correlation coefficient** | | | |
| --- | --- | --- | --- | --- |
|  | **Anorexia nervosa** | **Inflammatory bowel disease** | **Crohn's disease** | **Ulcerative colitis** |
| *p* parameter |  |  |  |  |
| LDpred-inf | 0.11321 | 0.00051 | 0.01063 | -0.00567 |
| 1 | 0.11347 | 0.00114 | -0.0107 | -0.00374 |
| 0.3 | 0.11502 | 0.00465 | -0.00866 | -0.01094 |
| 0.1 | 0.11682 | 0.01236 | -0.0016 | -0.00808 |
| 0.03 | 0.11021 | 0.00583 | 0.00483 | -0.00862 |
| 0.01 | 0.0926 | 0.0055 | 0.00956 | -0.00113 |
| 0.003 | 0.06632 | 0.00547 | 0.01192 | 0.00295 |
| 0.001 | 0.04123 | 0.00347 | 0.01207 | 0.00598 |

Table S3. Association between outcome diagnoses and covariates. Hazard ratios (HR) with 95% confidence intervals (CI).

|  | **Exposure diagnosis** | **Outcome diagnosis** | **Covariate** | **HR (95% CI)** | **p** |
| --- | --- | --- | --- | --- | --- |
| Within-individual diagnoses | Prior anorexia nervosa | Inflammatory bowel disease | Parental education level |  |  |
|  |  |  | Both basic school | 1.00 (ref) |  |
|  |  |  | Highest level vocational training/high school | 1.07 (0.99,1.15) | 0.071 |
|  |  |  | Highest level short- or medium-term | 1.00 (0.93,1.09) | 0.935 |
|  |  |  | Highest level long-term higher education | 0.90 (0.81,0.99) | 0.035 |
|  |  |  | Birth year (quartiles) |  |  |
|  |  |  | 1 | 1.00 (ref) |  |
|  |  |  | 2 | 1.18 (1.12,1.26) | <0.001 |
|  |  |  | 3 | 1.29 (1.19,1.40) | <0.001 |
|  |  |  | 4 | 1.62 (1.39,1.89) | <0.001 |
|  |  |  | Sex |  |  |
|  |  |  | Female | 1.00 (ref) |  |
|  |  |  | Male | 0.78 (0.74,0.82) | <0.001 |
|  |  |  | Age at exposure diagnosis (per year) | 0.98 (0.92,1.04) | 0.421 |
|  | Prior anorexia nervosa | Crohn's disease | Parental education level |  |  |
|  |  |  | Both basic school | 1.00 (ref) |  |
|  |  |  | Highest level vocational training/high school | 0.98 (0.89,1.09) | 0.747 |
|  |  |  | Highest level short- or medium-term | 0.88 (0.78,0.98) | 0.019 |
|  |  |  | Highest level long-term higher education | 0.74 (0.63,0.85) | <0.001 |
|  |  |  | Birth year (quartiles) |  |  |
|  |  |  | 1 | 1.00 (ref) |  |
|  |  |  | 2 | 1.25 (1.15,1.36) | <0.001 |
|  |  |  | 3 | 1.50 (1.35,1.68) | <0.001 |
|  |  |  | 4 | 2.09 (1.71,2.56) | <0.001 |
|  |  |  | Sex |  |  |
|  |  |  | Female | 1.00 (ref) |  |
|  |  |  | Male | 0.76 (0.71,0.82) | <0.001 |
|  |  |  | Age at exposure diagnosis (per year) | 0.97 (0.89,1.06) | 0.491 |
|  | Prior anorexia nervosa | Ulcerative colitis | Parental education level |  |  |
|  |  |  | Both basic school | 1.00 (ref) |  |
|  |  |  | Highest level vocational training/high school | 1.12 (1.02,1.23) | 0.016 |
|  |  |  | Highest level short- or medium-term | 1.09 (0.98,1.21) | 0.098 |
|  |  |  | Highest level long-term higher education | 1.00 (0.88,1.14) | 0.966 |
|  |  |  | Birth year (quartiles) |  |  |
|  |  |  | 1 | 1.00 (ref) |  |
|  |  |  | 2 | 1.14 (1.05,1.22) | 0.001 |
|  |  |  | 3 | 1.18 (1.07,1.31) | 0.002 |
|  |  |  | 4 | 1.31 (1.06,1.63) | 0.012 |
|  |  |  | Sex |  |  |
|  |  |  | Female | 1.00 (ref) |  |
|  |  |  | Male | 0.80 (0.75,0.85) | <0.001 |
|  |  |  | Age at exposure diagnosis (per year) | 0.97 (0.90,1.04) | 0.413 |
|  | Prior inflammatory bowel disease | Anorexia nervpsa | Parental education level |  |  |
|  |  |  | Both basic school | 1.00 (ref) |  |
|  |  |  | Highest level vocational training/high school | 1.19 (1.08,1.31) | 0.001 |
|  |  |  | Highest level short- or medium-term | 1.48 (1.34,1.64) | <0.001 |
|  |  |  | Highest level long-term higher education | 1.89 (1.69,2.12) | <0.001 |
|  |  |  | Birth year (quartiles) |  |  |
|  |  |  | 1 | 1.00 (ref) |  |
|  |  |  | 2 | 1.27 (1.18,1.36) | <0.001 |
|  |  |  | 3 | 1.73 (1.60,1.87) | <0.001 |
|  |  |  | 4 | 1.97 (1.68,2.32) | <0.001 |
|  |  |  | Sex |  |  |
|  |  |  | Female | 1.00 (ref) |  |
|  |  |  | Male | 0.07 (0.06,0.07) | <0.001 |
|  |  |  | Age at exposure diagnosis (per year) | 1.02 (0.97,1.06) | 0.539 |
|  | Prior Crohn's disease | Anorexia nervosa | Parental education level |  |  |
|  |  |  | Both basic school | 1.00 (ref) |  |
|  |  |  | Highest level vocational training/high school | 1.19 (1.08,1.31) | 0.001 |
|  |  |  | Highest level short- or medium-term | 1.48 (1.34,1.64) | <0.001 |
|  |  |  | Highest level long-term higher education | 1.90 (1.69,2.12) | <0.001 |
|  |  |  | Birth year (quartiles) |  |  |
|  |  |  | 1 | 1.00 (ref) |  |
|  |  |  | 2 | 1.27 (1.18,1.36) | <0.001 |
|  |  |  | 3 | 1.73 (1.60,1.87) | <0.001 |
|  |  |  | 4 | 1.97 (1.68,2.32) | <0.001 |
|  |  |  | Sex |  |  |
|  |  |  | Female | 1.00 (ref) |  |
|  |  |  | Male | 0.07 (0.06,0.07) | <0.001 |
|  |  |  | Age at exposure diagnosis (per year) | 1.01 (0.94,1.08) | 0.778 |
|  | Prior ulcerative colitis | Anorexia nervosa | Parental education level |  |  |
|  |  |  | Both basic school | 1.00 (ref) |  |
|  |  |  | Highest level vocational training/high school | 1.19 (1.08,1.31) | 0.001 |
|  |  |  | Highest level short- or medium-term | 1.48 (1.34,1.64) | <0.001 |
|  |  |  | Highest level long-term higher education | 1.89 (1.69,2.12) | <0.001 |
|  |  |  | Birth year (quartiles) |  |  |
|  |  |  | 1 | 1.00 (ref) |  |
|  |  |  | 2 | 1.27 (1.18,1.36) | <0.001 |
|  |  |  | 3 | 1.73 (1.60,1.87) | <0.001 |
|  |  |  | 4 | 1.97 (1.68,2.32) | <0.001 |
|  |  |  | Sex |  |  |
|  |  |  | Female | 1.00 (ref) |  |
|  |  |  | Male | 0.07 (0.06,0.07) | <0.001 |
|  |  |  | Age at exposure diagnosis (per year) | 1.03 (0.97,1.09) | 0.324 |

Table S4. Association between within-individual diagnoses, depending on time since exposure diagnosis. Hazard ratios (HR) with 95% confidence intervals (CI)

|  | **Exposure diagnosis** | **Outcome diagnosis** | **Time since exposure diagnosis** | **HR (95% CI)** | **p** |
| --- | --- | --- | --- | --- | --- |
| Within-individual diagnoses | Prior anorexia nervosa | Inflammatory bowel disease | No diagnosis | 1.00 (ref) |  |
|  |  |  | 0-<1 year | 1.66 (0.69,4.00) | 0.256 |
|  |  |  | 1-<5 years | 1.24 (0.73,2.09) | 0.427 |
|  |  |  | >5 years | 1.57 (1.01,2.43) | 0.045 |
|  | Prior anorexia nervosa | Crohn's disease | No diagnosis | 1.00 (ref) |  |
|  |  |  | 0-<1 year | 2.62 (0.98,6.99) | 0.054 |
|  |  |  | 1-<5 years | 1.78 (0.96,3.32) | 0.068 |
|  |  |  | >5 years | 1.16 (0.55,2.45) | 0.691 |
|  | Prior anorexia nervosa | Ulcerative colitis | No diagnosis | 1.00 (ref) |  |
|  |  |  | 0-<1 year | 1.61 (0.52,5.00) | 0.409 |
|  |  |  | 1-<5 years | 1.41 (0.76,2.62) | 0.280 |
|  |  |  | >5 years | 1.88 (1.15,3.08) | 0.012 |
|  | Prior inflammatory bowel disease | Anorexia nervosa | No diagnosis | 1.00 (ref) |  |
|  |  |  | 0-<1 year | 2.53 (1.20,5.31) | 0.014 |
|  |  |  | 1-<5 years | 1.20 (0.62,2.30) | 0.589 |
|  |  |  | >5 years | 0.78 (0.29,2.09) | 0.628 |
|  | Prior Crohn's disease | Anorexia nervosa | No diagnosis | 1.00 (ref) |  |
|  |  |  | 0-<1 year | 1.42 (0.36,5.69) | 0.618 |
|  |  |  | 1-<5 years | 1.59 (0.72,3.55) | 0.254 |
|  |  |  | >5 years | N/A |  |
|  | Prior ulcerative colitis | Anorexia nervosa | No diagnosis | 1.00 (ref) |  |
|  |  |  | 0-<1 year | 2.90 (1.21,6.96) | 0.017 |
|  |  |  | 1-<5 years | 0.63 (0.20,1.97) | 0.431 |
|  |  |  | >5 years | 1.29 (0.48,3.43) | 0.614 |

Table S5. Associations between polygenic scores (PGS) and diagnosis of the same disorder. Odds ratios (OR) with 95% confidence intervals (CI) per one standard deviation (SD) increase in PGS.

| **Exposure PGS** | **Outcome diagnosis** | **OR (95% CI)/SD** | **p** | **Nagelkerke pseudo-R2** | | |
| --- | --- | --- | --- | --- | --- | --- |
|  |  |  |  | **Null model** | **Full model** | **Difference** |
| Anorexia nervosa | Anorexia nervosa | 1.38 (1.32,1.44) | <0.001 | 0.231002 | 0.247269 | 0.016267 |
| Inflammatory bowel disease | Inflammatory bowel disease | 1.00 (0.80,1.26) | 0.996 | 0.1977212 | 0.197214 | 0 |
| Crohn's disease | Crohn's disease | 1.27 (0.93,1.73) | 0.138 | 0.110876 | 0.119192 | 0.008315 |
| Ulcerative colitis | Ulcerative colitis | 1.12 (0.83,1.52) | 0.445 | 0.189134 | 0.191595 | 0.002461 |
| Depression | Depression | 1.33 (1.29,1.37) | <0.001 | 0.328959 | 0.353223 | 0.024274 |

Table S6. Results from the Mendelian randomization analyses

|  |  |  | **MR-Egger** | | | **MR-Egger intercept** | | | **MR-Egger heterogeneity test** | | |
| --- | --- | --- | --- | --- | --- | --- | --- | --- | --- | --- | --- |
| **Exposure** | **Outcome** | **SNPs** | **Slope** | **Standard error** | **p** | **Intercept** | **Standard error** | **p** | **Q** | **Q_df** | **Q_p** |
| Anorexia nervosa | Inflammatory bowel diseases | 4 | -0.902 | 0.688 | 0.320 | 0.060 | 0.058 | 0.409 | 0.796 | 2 | 0.672 |
| Anorexia nervosa | Crohn's disease | 4 | -1.395 | 0.884 | 0.255 | 0.087 | 0.074 | 0.363 | 0.160 | 2 | 0.923 |
| Anorexia nervosa | Ulcerative colitis | 4 | -0.017 | 0.908 | 0.987 | -0.005 | 0.076 | 0.951 | 2.128 | 2 | 0.345 |
| Inflammatory bowel diseases | Anorexia nervosa | 101 | 0.010 | 0.034 | 0.759 | -0.001 | 0.004 | 0.785 | 112.010 | 99 | 0.175 |
| Crohn's disease | Anorexia nervosa | 78 | -0.060 | 0.033 | 0.075 | 0.008 | 0.006 | 0.129 | 106.870 | 76 | 0.011 |
| Ulcerative colitis | Anorexia nervosa | 53 | 0.036 | 0.052 | 0.493 | -0.005 | 0.008 | 0.498 | 78.792 | 51 | 0.008 |
| Depression | Inflammatory bowel diseases | 4 | 0.550 | 0.665 | 0.495 | -0.034 | 0.034 | 0.557 | 1.428 | 2 | 0.490 |
| Depression | Crohn's disease | 4 | 1.120 | 0.862 | 0.299 | -0.054 | 0.044 | 0.349 | 2.004 | 2 | 0.367 |
| Depression | Ulcerative colitis | 4 | 0.043 | 0.843 | 0.964 | -0.001 | 0.043 | 0.992 | 0.552 | 2 | 0.759 |
| Inflammatory bowel diseases | Depression | 102 | -0.038 | 0.020 | 0.064 | 0.003 | 0.002 | 0.286 | 112.980 | 100 | 0.177 |
| Crohn's disease | Depression | 78 | -0.012 | 0.019 | 0.517 | 0.000 | 0.003 | 0.948 | 91.765 | 76 | 0.105 |
| Ulcerative colitis | Depression | 50 | -0.029 | 0.032 | 0.379 | 0.003 | 0.005 | 0.504 | 76.365 | 48 | 0.006 |
